# Recognizing and Differentiating Area Postrema Syndrome Across NMOSD, MOGAD, and GFAP Astrocytopathy

**DOI:** 10.3390/ijms27198661

**Published:** 2026-09-28

**Authors:** Eslam Shosha, Jihan Madani

**Affiliations:** 1 Neuroscience Center of Excellence, King Faisal Specialist Hospital and Research Center, MBC 76, Riyadh 11211, Saudi Arabia; jalyanbuaawy@kfshrc.edu.sa; 2 Neurology Division, Department of Medicine, McMaster University, Hamilton, ON L8L 2X2, Canada

**Keywords:** area postrema syndrome, neuromyelitis optica spectrum disorder, MOGAD, GFAP-astrocytopathy, glucagon-like peptide-1, aquaporin-4-IgG, gut–brain axis, complement activation

## Abstract

Area postrema syndrome (APS)—intractable nausea, vomiting, and/or hiccups persisting ≤48 h—is a recognized presenting feature of neuromyelitis optica spectrum disorder (NMOSD) and, less commonly, of myelin oligodendrocyte glycoprotein antibody-associated disease (MOGAD) and glial fibrillary acidic protein (GFAP)-astrocytopathy. APS is still frequently misattributed to primary gastrointestinal disease, delaying immunotherapy by weeks to months. For years its pathophysiology was inferred largely from lesion topography rather than demonstrated at the level of neuronal signaling. A study presented at the 2025 American Neurological Association annual meeting, and subsequently posted as a preprint, proposes a mechanistic account: elevated glucagon-like peptide-1 (GLP-1) and its receptor (GLP-1R) correlate with APS presence and severity in AQP4-IgG-positive NMOSD, and receptor blockade normalizes neuronal hyperexcitability in animal models. This finding comes from a single center and has not yet completed independent replication or full peer review; throughout this review we treat it as hypothesis-generating rather than established. Using that hypothesis as an organizing frame, this narrative review summarizes the anatomical and clinical basis of APS; expands the biological and clinical comparison of APS across NMOSD, MOGAD, and GFAP-astrocytopathy, including the mechanistic reasons the three disorders diverge; and characterizes the diagnostic pathway that routes patients through gastroenterology before neurology. It closes with the clinical implications, and the explicit limitations, of a putatively hormone-modulated area postrema circuit.

## 1. Introduction

Area postrema syndrome refers to acute or subacute episodes of intractable nausea, vomiting, or hiccups, occurring singly or in combination, that last at least 48 h and are not explained by another cause. Case-level recognition of intractable vomiting and hiccups as a feature of neuromyelitis optica dates back well over a decade. Early reports described it as a herald symptom preceding overt exacerbations [1]. Its status as a distinct, definable clinical entity was strengthened considerably by a 2011 neuropathological study that examined archival brainstem tissue from patients with neuromyelitis optica, multiple sclerosis, and neurologically normal controls. Lesions at the level of the area postrema and dorsal medullary floor were found selectively in the neuromyelitis optica group, and their presence was statistically associated with a documented history of intractable nausea, vomiting, or hiccups—direct histopathological support for what had previously been an anatomically plausible but unproven association [2].

This anatomical vulnerability is itself well precedented outside the autoimmune literature. The area postrema functions physiologically as the brain’s principal chemoreceptor trigger zone. It is a circumventricular organ on the dorsal medullary floor, built from glia and neurons covered by a thin ependymal layer and penetrated by fenestrated, non-tight-junctioned capillaries, which lets it sample circulating toxins, drugs, and hormones and relay that information to the medullary vomiting center [3]. Long before autoimmune demyelinating disease was implicated, neurosurgical case series had already shown that discrete lesions of the area postrema, of essentially any cause, could produce intractable vomiting [4]. This underscores that the vulnerability is structurally determined by the region itself rather than specific to any one disease process.

APS was recognized early as a distinctive presenting or relapsing feature of AQP4-IgG-positive NMOSD. This reflects the area postrema’s incomplete blood–brain barrier together with unusually dense AQP4 expression on astrocytic end-feet, which appears to make the region a preferential site of antibody- and complement-mediated astrocytic injury in this disease [5]. Formal diagnostic criteria and a severity scale, derived from an international multicenter database of 430 patients and validated in a prospective cohort, established that isolated APS attacks occur in roughly 7–10% of patients at onset and 9–15% over the disease course. These figures have anchored clinical suspicion for over a decade [6].

Occasionally the syndrome extends beyond the classical nausea–vomiting–hiccup triad. Because the area postrema sits within a broader dorsal medullary and vagal autonomic network, some patients develop syncope or other autonomic disturbance during an attack, reflecting a spread of dysfunction to adjacent brainstem structures rather than a separate process [7].

What has been missing until recently is a mechanistic account of why this particular anatomical vulnerability produces the specific symptom cluster of nausea, vomiting, and hiccups, rather than some other constellation of brainstem signs. A study presented at the 2025 American Neurological Association Annual Meeting, and subsequently posted as a preprint that has not yet completed formal peer review, reported that patients with AQP4-IgG-positive NMOSD and APS have elevated GLP-1 and GLP-1R levels in blood and cerebrospinal fluid compared with NMOSD patients without APS and with healthy controls. In animal models, this signaling was reported to drive glycolytic reprogramming and hyperexcitability in area postrema neurons, with symptom normalization upon receptor blockade [8]. This is, to our knowledge, the first mechanistic rather than purely topographic hypothesis for the syndrome, arriving more than a decade after the neuropathological case for the area postrema’s central role was first made [2]. Because this hypothesis derives from a single center and awaits independent replication, we treat it as hypothesis-generating throughout this review rather than as an established mechanism, and we return to this limitation explicitly in Section 8.

A subsequently published, independent case report described a patient who developed a seronegative NMOSD-like syndrome, including an area postrema lesion, coinciding with semaglutide use for weight loss [9]. A single case cannot establish causation, but it is a peer-reviewed data point independent of the original cohort, and it is discussed further, together with its limitations, in Section 7.4.

In parallel, a separate and largely disconnected line of work has characterized how APS differs across the three principal antibody-mediated demyelinating disorders in which it occurs: NMOSD, MOGAD, and GFAP-astrocytopathy. These studies report divergent frequencies and, notably, divergent lesion extent, but they have rarely been read together or connected to the emerging mechanistic data. A third thread, the tendency for APS to be misdiagnosed as primary gastrointestinal disease, has been documented but is rarely discussed as part of the same narrative as the mechanism or the cross-disorder comparison.

This review integrates these three threads, using the proposed GLP-1/GLP-1R mechanism as an organizing frame rather than an established fact: a candidate explanation for why the area postrema misfires, a possible reason its behavior might differ by underlying disease, and a lens for understanding why the syndrome is so often missed at first presentation.

## 2. Materials and Methods

This is a narrative review; no systematic review protocol was pre-registered. We conducted a directed search of PubMed/MEDLINE and preprint servers (medRxiv, Research Square) for English-language records on area postrema syndrome, NMOSD, MOGAD, GFAP-astrocytopathy, and GLP-1/GLP-1R, combining these terms with Boolean operators and supplementing the search by manually screening the reference lists of retrieved articles and recent reviews. The search was conducted through July 2026 and updated shortly before submission. Records were screened for relevance by the author; because this is a narrative rather than a systematic review, no dual independent screening, formal risk-of-bias assessment, or PRISMA-type flow diagram was performed, consistent with recommended reporting practice for narrative reviews. Beyond the original conference-presented GLP-1/GLP-1R dataset and its preprint, this update identified one additional independent, peer-reviewed data point (the case report discussed in Section 1 and Section 7.4) but no further primary study directly testing the GLP-1/GLP-1R hypothesis in APS. Because this review synthesizes qualitatively heterogeneous clinical, radiological, and mechanistic literature rather than pooling comparable quantitative outcomes, findings are presented thematically across Section 3, Section 4, Section 5, Section 6 and Section 7 rather than in a separate, stand-alone Results section, an approach consistent with current guidance for structuring narrative reviews [10].

## 3. Anatomical and Clinical Basis of APS

### 3.1. The Area Postrema as a Structurally Privileged, and Vulnerable, Site

The area postrema is one of several circumventricular organs lacking a complete blood–brain barrier. Under ordinary physiology, this property allows it to sample circulating signals, including gut hormones, toxins, and drugs, as part of its role in nausea, emesis, and satiety regulation, precisely why it is classically termed the chemoreceptor trigger zone [3]. In AQP4-IgG-positive NMOSD, this same permeability appears to make the region a preferential entry point for pathogenic antibody and complement-mediated astrocytic injury, given the unusually high density of AQP4 water channels on astrocytic end-feet there. This pattern is also reflected in the tendency of NMOSD brain lesions more generally to localize to other high-AQP4-expression sites such as the periventricular diencephalon, hypothalamus, and periaqueductal grey matter [5].

Mechanistically, AQP4-IgG is an IgG1 antibody capable of fixing complement. Binding to AQP4 tetramers clustered in orthogonal arrays on the astrocytic end-foot membrane activates the classical complement cascade, from C1q engagement through formation of the membrane attack complex, producing direct astrocytic lysis as well as recruitment of granulocytes and eosinophils that amplify tissue injury [11]. Astrocyte loss in turn compromises the glial support of endothelial tight junctions, so the same lesion that begins as antibody-mediated astrocytopathy also degrades local blood–brain barrier integrity, a self-reinforcing cycle that both permits greater antibody and complement access and may help create the locally inflamed tissue environment in which the GLP-1/GLP-1R signal described in Section 4 has been proposed to arise [11]. Because the area postrema already has an incomplete barrier under normal physiology, this superimposed pathological compromise plausibly lowers the threshold at which circulating and locally produced GLP-1 can act on GLP-1R-expressing neurons there, though this specific link has not been directly demonstrated and remains inferential rather than established.

Neuropathological correlation strengthens the causal interpretation of this anatomical association. In a 2011 study by Popescu et al., roughly forty percent of patients with confirmed neuromyelitis optica had qualifying lesions at the area postrema/dorsal medullary floor [2]. The presence of these lesions was associated with significantly increased odds of a clinical history of nausea, vomiting, or hiccups, a link present in neither the multiple sclerosis comparison group nor normal controls [2]. This provided direct tissue-level evidence that the symptom is not coincidental to disease location but plausibly caused by focal injury at this site.

### 3.2. Diagnostic Criteria and Clinical Texture

The Shosha et al. (2018) criteria operationalized APS as intractable nausea, vomiting, or hiccups (singly or combined) persisting at least 48 h and unexplained by another etiology, paired with a severity scale adapted from a nausea-and-emesis quantification tool originally developed in obstetric care (the Pregnancy-Unique Quantification of Emesis and Nausea instrument) [6]. Applying these criteria to a large international NMOSD dataset, roughly seven to ten percent of patients presented with APS as an isolated onset attack, with slightly higher subsequent-attack frequencies. This pattern held consistently across continents, arguing against a purely regional or ascertainment-based artifact [6]. Within the validation cohort, most patients experienced a single APS attack over their disease course, while a minority went on to have multiple recurrent episodes [6].

Radiologically, APS attacks typically correspond to a T2/FLAIR-hyperintense, sometimes a gadolinium-enhancing, lesion involving the dorsal medulla at the level of the area postrema and fourth ventricle, occasionally extending into the adjacent nucleus tractus solitarius or cervicomedullary junction. This lesion pattern has long served as the radiological correlate that lends the clinical syndrome its name, but until 2025 it explained localization without explaining the mechanism.

## 4. A Proposed, Single-Center Mechanism: GLP-1/GLP-1R Signaling in the Area Postrema

### 4.1. GLP-1 Physiology as Background

Before considering its proposed role in APS, it is useful to recall GLP-1’s ordinary physiology. GLP-1 is a 30-amino-acid peptide produced mainly by intestinal L-cells and released after meals. It is rapidly degraded in circulation, so many of its physiological actions on appetite, gastric emptying, and nausea signaling are thought to be relayed not by the hormone reaching the brain intact, but via vagal sensory afferents terminating in the nucleus tractus solitarius (NTS) and the neighboring area postrema, both components of the dorsal vagal complex [12] (Figure 1). Beyond relaying gut-derived signals, preproglucagon-expressing neurons have also been identified within the NTS itself, providing a potential source of centrally produced GLP-1 that is anatomically distinct from intestinal L-cell hormone, and GLP-1 receptors are expressed on neurons in both the area postrema and NTS; the relative physiological contribution of this local, centrally produced pool versus vagally relayed gut-derived GLP-1 to normal satiety and emesis signaling is not fully resolved and remains an area of ongoing investigation [12]. This dual gut–vagal and central circuitry is the physiological substrate that the 2025 findings propose is pathologically co-opted in NMOSD. It is not a novel target invented for this disease, which is part of why the hypothesis is biologically plausible even though direct evidence in APS remains limited to one dataset.

### 4.2. The Proposed 2025/2026 Finding

The 2025 ANA-presented study by Yang and colleagues, now available as a preprint that has not completed peer review, measured GLP-1 and GLP-1R levels in blood and CSF from 248 patients with AQP4-IgG-positive NMOSD, 57 of whom had a history of APS, alongside 164 healthy controls [8,13]. Both molecules were reported as substantially higher in the APS group than in NMOSD patients without APS or in controls, with higher levels tracking greater clinical severity. Levels reportedly declined following immunotherapy, consistent with a state marker of active disease rather than a fixed trait, though this remains an uncontrolled, single-center observation that requires replication before it can be interpreted as a validated biomarker [8].

In parallel animal experiments, using a dual-antigen (AQP4 and MOG) experimental autoimmune encephalomyelitis model together with SH-SY5Y neuronal cultures, mice with APS-like symptoms showed increased GLP-1 and GLP-1R expression in area postrema neurons. This was associated with a pattern the authors describe as glycolytic reprogramming, assessed using non-targeted metabolomics, patch-clamp electrophysiology, and transmission electron microscopy [8]. Pharmacological GLP-1R blockade with exendin-(9-39) normalized this neuronal activity and resolved the APS-like behavioral phenotype [8]. The authors additionally reported that GLP-1R agonism could provoke abnormal excitability elsewhere in the dorsal vagal complex, including the NTS, suggesting the proposed circuit may not be confined to the area postrema alone [8].

Several aspects of this preclinical model warrant explicit caveat. First, the “APS-like” phenotype in mice was operationalized primarily as reduced food intake and impaired body-weight gain, parameters that are physiologically nonspecific and can result from pain, general illness behavior, motor impairment, or numerous causes unrelated to area-postrema-mediated nausea; they are not direct behavioral equivalents of the persistent nausea, vomiting, and/or hiccups that define human APS, and rodents lack a vomiting reflex altogether, which limits how directly murine behavioral readouts can be mapped onto the human syndrome. Second, the experimental autoimmune encephalomyelitis model used a dual-antigen (AQP4 and MOG) immunization protocol, which introduces a demyelinating and inflammatory component associated with MOG immunization that is not present in typical AQP4-IgG-positive human NMOSD and is not, by itself, a validated model of spontaneous AQP4-antibody-mediated astrocytopathy. Third, the in vitro work used SH-SY5Y neuroblastoma-derived cells, a widely used and tractable neuronal model but one that is not derived from the area postrema and does not reproduce the specific neuron–glia interactions, fenestrated vasculature, or circumventricular microenvironment of the dorsal vagal complex described in Section 3.1. These are useful, hypothesis-generating experimental systems, but each represents a step removed from the human disease and clinical phenotype they are intended to model, and this gap should be weighed when interpreting the strength of the preclinical evidence summarized above.

Taken together, the human correlative data and the animal interventional data are consistent with a model in which local GLP-1/GLP-1R signaling, amplified in the context of astrocytic and neuroinflammatory injury, contributes to the hyperexcitability that produces the clinical syndrome, rather than the syndrome arising simply as a passive consequence of lesion location. We emphasize, however, that this model rests on a single, not-yet-peer-reviewed dataset. It requires independent confirmation, ideally in a separate cohort with pre-specified thresholds, before it can be considered an established disease mechanism rather than a plausible hypothesis.

This hypothesis is biologically plausible on independent grounds, since, as noted in Section 4.1, the area postrema is already a known physiological target of circulating and vagally relayed GLP-1 as part of normal appetite and nausea signaling. A structure already wired to respond to this hormone is a reasonable, though unproven, substrate for pathological amplification once local inflammation and blood–brain barrier compromise are present. If confirmed, the finding would reframe APS from a lesion that happens to sit in a symptom-relevant location to a circuit whose native hormonal sensitivity is being pathologically exploited, with implications for biomarkers and symptomatic therapy discussed in Section 7, Section 8 and Section 9. We stress “if confirmed” deliberately: this reframing is not yet established.

Figure 2 summarizes this proposed cascade as a single working model, with each element labeled by its current evidentiary status: astrocytic injury and blood–brain barrier compromise at the area postrema (established) are proposed to lead to local GLP-1/GLP-1R elevation (preliminary, single-center), which is proposed to drive glycolytic reprogramming, neuronal hyperexcitability, and possible spread along the dorsal vagal complex (preliminary), producing the clinical syndrome. GLP-1R blockade interrupted this cascade in animal models, while exogenous GLP-1 receptor agonism is flagged as a hypothesized, unproven amplifying input, discussed further in Section 7.

### 4.3. Relationship to Classical Emetic Neurotransmitter Systems

The GLP-1/GLP-1R hypothesis should be situated within, rather than in place of, the established neuropharmacology of nausea and emesis. Area postrema and nucleus tractus solitarius neurons integrate multiple convergent inputs beyond GLP-1, including dopaminergic transmission at the chemoreceptor trigger zone acting on D2 receptors, serotonergic signaling from enterochromaffin cells and vagal afferents acting on 5-HT3 receptors, substance P and neurokinin-1 (NK1) receptor signaling in the nucleus tractus solitarius, and endocannabinoid modulation of vagal and area postrema excitability. These pathways are precisely the targets of the antiemetic drugs used in current practice (dopamine antagonists, 5-HT3 antagonists, and NK1 antagonists such as aprepitant). Whether AQP4-IgG-mediated astrocytic injury and the proposed GLP-1/GLP-1R signal act in parallel with, upstream of, or convergently onto these classical circuits is not established; glycolytic reprogramming and neuronal hyperexcitability, as described in Section 4.2, could plausibly lower the threshold at which dopaminergic, serotonergic, or substance-P-mediated inputs trigger emesis, rather than acting as an independent trigger. This convergence has a direct clinical corollary, discussed further in Section 7.3: if GLP-1R antagonism proves useful, it is more likely to function as an adjunct that raises the threshold for activation of these downstream classical pathways than as a stand-alone replacement for dopamine-, serotonin-, or NK1-receptor-targeted antiemetics.

## 5. APS Across the Spectrum: NMOSD, MOGAD, and GFAP-Astrocytopathy

NMOSD (AQP4-IgG-positive) remains both the most frequent host disease for APS and the only one in which the GLP-1/GLP-1R mechanism has so far been proposed. Onset and subsequent-attack frequencies of roughly 7–15% (Section 3) make APS a common enough presenting feature that isolated, unexplained intractable nausea, vomiting, or hiccups should prompt AQP4-IgG testing even in the absence of optic neuritis or myelopathy [6].

MOGAD presents a more heterogeneous picture. A multicenter Korean cohort comparing adults with AQP4 versus MOG antibodies found APS at onset in 14.9% of AQP4-positive patients versus none of the MOG-antibody patients, and during the disease course in 17.2% versus 1.9% respectively. This highly significant difference, together with earlier pediatric and adult European data showing similarly low MOG-antibody frequencies, has generally supported the view that APS is a comparatively AQP4-selective phenomenon (Figure 3) [14].

Biologically, this divergence in frequency is consistent with the differing immunopathology of the two diseases. MOG-IgG targets myelin oligodendrocyte glycoprotein on the oligodendrocyte and myelin sheath rather than the astrocytic AQP4 water channel, and MOGAD lesions are thought to arise through a combination of antibody-dependent and T-cell-mediated demyelination, with comparatively less reliance on complement-mediated astrocytic lysis than AQP4-positive NMOSD [14,15,16]. Because the area postrema’s vulnerability in NMOSD is mechanistically linked to its unusually dense astrocytic AQP4 expression, a disease process that spares AQP4 as its primary target would be expected, on structural grounds alone, to involve the area postrema less consistently. This offers a plausible biological explanation for MOGAD’s lower and more variable APS frequency, rather than this pattern being merely a statistical artifact of small cohorts.

This apparent AQP4-selectivity is not uniform across all cohorts, however. At least one cohort, a Caucasian, adult-predominant MOG-antibody series, reported a notably higher APS frequency of roughly 15% [15]. A UAE-based comparison of MOGAD and NMOSD patients found brainstem syndrome with APS more common in the MOGAD cohort than in NMOSD (29% versus 4%), a striking reversal of the more typical pattern shown in Figure 3 [17]. Reasons for this heterogeneity, rather than a true biological reversal, are discussed below.

Beyond frequency, a 2024 comparative study examining lesion topography in APS attacks found that when APS does occur in MOGAD, it is associated with significantly more extensive disease. Brainstem regional involvement and involvement of other brain regions were both markedly more common in MOGAD-associated APS attacks than in NMOSD-associated attacks [18]. This suggests that even though APS is individually rarer in MOGAD, when it occurs it may reflect a more diffusely inflammatory process than the comparatively localized, area-postrema-centered lesion typical of AQP4-positive NMOSD. This is a distinction with plausible mechanistic significance, since the GLP-1/GLP-1R axis described above has only been characterized in the NMOSD context and may not generalize to a more widespread MOGAD-associated encephalitic process.

GFAP-astrocytopathy is the most recently characterized host disease for APS, and it differs mechanistically from both NMOSD and MOGAD. The pathogenic antibody targets glial fibrillary acidic protein, an intracellular intermediate filament protein rather than a cell-surface antigen, so the disease is generally considered a T-cell-mediated meningoencephalomyelitis with corticosteroid-responsive inflammation rather than a complement-fixing, antibody-mediated cytotoxic process [19,20]. This distinction matters clinically as well as mechanistically: GFAP-astrocytopathy typically also produces a CSF lymphocytic pleocytosis, linear perivascular gadolinium enhancement, and a meningoencephalitic clinical picture, including headache, encephalopathy, and tremor, that can accompany or overshadow any co-existing area postrema involvement, in contrast to the comparatively isolated brainstem syndrome more typical of AQP4-positive NMOSD [19,20].

A 2025 case series and literature comparison found APS to be a comparatively uncommon presenting feature of autoimmune GFAP-IgG-positive astrocytopathy relative to AQP4-positive NMOSD [19]. A separate report described MRI features, including linear leptomeningeal-type enhancement along the brainstem and fourth ventricle surface, that can help distinguish GFAP-astrocytopathy-associated APS from the more classic area postrema parenchymal lesion of NMOSD [20]. Because the mechanism proposed in Section 4 is specifically tied to AQP4-driven astrocytic injury, whether an analogous GLP-1/GLP-1R signal exists in an intracellular-antigen, T-cell-predominant disease like GFAP-astrocytopathy is, at minimum, mechanistically uncertain rather than a safe extrapolation.

Read together, these three disorders share a final common clinical pathway—intractable nausea, vomiting, or hiccups arising from dorsal medullary/area postrema involvement—but diverge in immunopathology, frequency, and typical lesion extent. Table 1 summarizes these points of convergence and divergence, and Figure 4 illustrates the distinguishing MRI patterns summarized in its "imaging clue" row. Whether the GLP-1/GLP-1R axis operates in MOGAD- or GFAP-astrocytopathy-associated APS, or whether their distinct immunopathology and more diffuse lesion pattern point to an entirely different driver, is an open and clinically consequential question rather than a settled extrapolation from the NMOSD data.

The GLP-1/GLP-1R mechanism row above reflects a single, not-yet-peer-reviewed dataset generated in AQP4-IgG-positive NMOSD only (Section 4.2); it has not been tested in MOGAD or GFAP-astrocytopathy and should not be read as an established or disorder-general feature of area postrema syndrome. Frequency figures throughout this table should likewise be read as estimates from heterogeneous, largely unreplicated cohorts (Section 5.1) rather than precise population rates.

### 5.1. Why Do Frequency Estimates Conflict? A Critical Synthesis

The frequency estimates summarized in Table 1, and the apparent reversal reported in the UAE-based cohort, are best read as a heterogeneous literature rather than a single settled figure. Several methodological factors plausibly explain this heterogeneity rather than a true biological reversal in every population.

First, most series are small, retrospective, and single-center, so point estimates of an already uncommon presentation, APS in MOGAD, carry wide and often unreported confidence intervals and are sensitive to a handful of cases. Second, antibody-testing platforms differ across centers and eras: live cell-based assays for MOG-IgG and AQP4-IgG have different sensitivity and specificity profiles than older fixed cell-based or ELISA methods, so cohorts assembled before live-assay adoption may include misclassified patients. Third, imaging protocols vary in slice thickness, sequence choice, and whether dedicated brainstem sequences were obtained; these factors directly affect whether a small-area postrema lesion is detected at all. Fourth, case ascertainment and the operational definition of APS have not been fully harmonized across studies: some apply the formal Shosha et al. criteria, others rely on chart-review-based symptom capture, which can shift apparent frequency in either direction. Finally, cohort ethnicity and regional referral patterns differ substantially, from Korean and Japanese series to Caucasian European cohorts to the UAE comparison, and both NMOSD and MOGAD show some population-level variation in phenotype, so frequency estimates from one region cannot be assumed to generalize to another without confirmation.

None of this invalidates the broad pattern that APS is more consistently frequent in AQP4-positive NMOSD than in MOGAD or GFAP-astrocytopathy. It does mean, however, that the individual percentage figures in Table 1 should be read as estimates from heterogeneous, largely unreplicated cohorts rather than precise, generalizable rates.

### 5.2. Pediatric APS: A Note on Scope

The data reviewed above are drawn overwhelmingly from adult cohorts, and pediatric APS is not addressed as a separate topic in this review. This is a meaningful gap: MOGAD is proportionally far more common than AQP4-IgG-positive NMOSD in children, the reverse of the adult pattern described above, so a review organized primarily around AQP4-selective mechanisms risks under-representing the pediatric-relevant disease. The differential diagnosis of intractable vomiting or hiccups in children also differs materially from that in adults, including cyclic vomiting syndrome, mitochondrial disease, and other pediatric-specific gastrointestinal and metabolic disorders that are uncommon considerations in adult practice. Whether the GLP-1/GLP-1R signal described in Section 4, characterized so far only in an adult AQP4-IgG-positive cohort, applies to pediatric NMOSD or to MOGAD, the more relevant pediatric disease, is untested and should not be assumed. We therefore state explicitly that pediatric APS falls outside the scope of this review, and flag this as a limitation (Section 8) rather than treat the adult-derived framework as directly transferable to children.

## 6. Diagnostic Pitfalls: The Gastroenterology Detour

### 6.1. Documented Pattern of Misdiagnosis

A recurring and clinically costly feature of APS is that it is frequently misread as primary gastrointestinal disease before it is recognized as neurological. In the cohort underlying the 2018 diagnostic criteria, forty-four patients had presented initially to gastroenterologists and undergone extensive evaluation, including upper endoscopy, gastric transit studies, and abdominal CT, without an identified cause. Roughly one in five of these patients was given an incorrect diagnosis, including gastroparesis, food poisoning, gastroesophageal reflux disease, Helicobacter pylori infection, cholecystitis, Crohn disease, pancreatitis, fatty liver, Clostridium difficile infection, or a psychogenic attribution [6]. Independent case series from other regions describe an essentially identical pattern of extensive, unrevealing gastrointestinal workup preceding eventual neurological diagnosis, suggesting this is a structural feature of how the syndrome is triaged rather than an artifact of any single health system.

This detour has a direct clinical cost: time spent pursuing a gastrointestinal explanation is time without immunotherapy, during which relapse-associated axonal and astrocytic injury can accrue, which is particularly consequential in a disease where a single severe attack can produce permanent disability. Several factors likely sustain this pattern: the intuitive framing of vomiting and hiccups as digestive symptoms, the absence of the more instantly recognizable visual or spinal cord findings that typically trigger neurological referral, and limited awareness of APS as a distinct neuroimmunological entity outside specialized demyelinating-disease centers.

### 6.2. A New Risk Introduced by the GLP-1 Hypothesis

The emerging GLP-1 hypothesis introduces a subtle new risk of reinforcing, rather than correcting, this pattern. Because GLP-1 is a gut-derived hormone most familiar to clinicians in the context of diabetes and appetite regulation, an incomplete reading of the new mechanistic data could be mistaken for evidence pointing back toward a gastrointestinal or metabolic explanation, when in fact the finding describes a proposed central neuroinflammatory circuit being modulated by that hormone, not a peripheral digestive process. Communicating the hypothesis precisely, as a preliminary, central, disease-specific neuronal phenomenon rather than an established peripheral endocrine one, is therefore itself part of closing the diagnostic gap. This is a point we deliberately foreground rather than treat as an incidental footnote, and it applies equally to the case-level signal discussed in Section 7.4.

### 6.3. A Practical Recognition Heuristic

Across the disorders reviewed here, three features recur as red flags that should prompt neurological and serological evaluation before, or alongside, gastrointestinal workup: symptom duration meeting or exceeding 48 h; a negative or unrevealing standard gastrointestinal evaluation (endoscopy, transit studies, imaging); and any accompanying, even subtle, neurological symptom, (diplopia, gait change, sensory disturbance, or unexplained syncope), that might otherwise be dismissed as unrelated. None of these are individually diagnostic, but their combination should lower the threshold for AQP4-IgG and MOG-IgG testing substantially below what typical gastroenterology practice would otherwise apply.

## 7. Therapeutic and Clinical Implications

### 7.1. Treating the Underlying Relapse: The Therapeutic Priority

When APS occurs as a manifestation of an active NMOSD, MOGAD, or GFAP-astrocytopathy relapse, as is the case for the great majority of the attacks discussed throughout this review, the therapeutic priority is rapid treatment of the underlying inflammatory process, not symptomatic control of nausea, vomiting or hiccups in isolation. Current relapse-management principles for AQP4-IgG-positive NMOSD center on high-dose intravenous glucocorticoids as first-line therapy, with early escalation to plasma exchange (plasmapheresis) or immunoadsorption for attacks that respond incompletely or in patients with more severe presentations, since more rapid initiation of apheresis has been associated with better attack-related outcomes in NMOSD; analogous principles of prompt high-dose steroid treatment, with escalation to apheresis for refractory attacks, are generally applied to relapses of MOGAD and GFAP-astrocytopathy, informed by their own respective evidence bases. Antiemetic therapy, whether with existing dopamine- or 5-HT3-receptor antagonists or, prospectively, with the GLP-1R-targeted approach discussed in Section 7.3, should be understood as supportive and symptomatic, valuable for patient comfort and for bridging the interval before immunotherapy takes effect, but not as a substitute for prompt disease-modifying treatment of the underlying relapse.

### 7.2. Immediate Diagnostic Implications

At the bedside, the practical implication is straightforward: an unexplained episode of intractable nausea, vomiting, or hiccups lasting 48 h or longer, particularly one that has already undergone a negative gastroenterological workup, should prompt AQP4-IgG and MOG-IgG serological testing and dedicated MRI sequences through the area postrema and dorsal medulla, even in the absence of classical optic neuritis or myelitis. Given the divergent lesion extent summarized in Table 1, imaging protocols should not stop at the area postrema itself when MOGAD is suspected, since disease may extend more broadly through the brainstem and beyond.

Because nausea and vomiting are extremely common symptoms while NMOSD and MOGAD are rare, this threshold should not be applied indiscriminately to all prolonged nausea or vomiting; as detailed in Section 6.3, testing is best reserved for patients who additionally meet at least one further red flag—an unrevealing standard gastrointestinal evaluation and/or any accompanying, even subtle, neurological symptom—rather than for symptom duration alone.

### 7.3. A Candidate, Hypothesis-Generating Therapeutic Target

Mechanistically, the preliminary demonstration that GLP-1R blockade normalizes neuronal hyperactivity and resolves APS-like symptoms in animal models [8] raises the possibility of a targeted, symptom-specific intervention distinct from disease-modifying immunotherapy, potentially useful as a bridge during acute attacks while immunosuppressive treatment takes effect. We emphasize that this remains an early-stage, single-center preclinical observation. It requires independent replication, human proof-of-concept, dose-finding, and safety data before any clinical extrapolation, and should currently be regarded as hypothesis-generating rather than a validated therapeutic strategy. Existing antiemetic regimens (e.g., dopamine antagonists, 5-HT3 antagonists) remain the current standard of symptomatic care pending such data.

Exendin-(9-39) is a 31-amino-acid peptide GLP-1R competitive antagonist derived by truncation of exendin-4; it has no meaningful agonist activity and displaces both endogenous GLP-1 and exogenous GLP-1R agonists from the receptor [21]. It has been administered to humans in mechanistic studies of post-bariatric and congenital hyperinsulinemic hypoglycemia [22,23], where intravenous and subcutaneous dosing raised fasting and post-prandial glucose and was generally well tolerated in short-term studies, providing some human safety experience, albeit outside the NMOSD/APS context and typically over hours to days rather than the sustained dosing that a therapeutic APS indication would likely require. As a peptide, exendin-(9-39) does not cross the blood–brain barrier efficiently under normal physiology [24]; its efficacy in the animal APS model presumably reflects action at the area postrema, one of the few brain regions accessible to circulating peptides precisely because of the incomplete barrier discussed in Section 3.1 [25], rather than broad CNS penetration. This is mechanistically reassuring for an area-postrema-selective application but also means efficacy could be lost if barrier integrity is restored by successful immunotherapy, and it underscores that formal pharmacokinetic and dose-finding studies specific to this route of action are still needed before any clinical extrapolation to APS.

### 7.4. A Cautionary, Hypothesis-Generating Signal Regarding Exogenous GLP-1 Receptor Agonists

A further, more immediate consideration follows from the rapid expansion of GLP-1 receptor agonist prescribing for diabetes and obesity. The authors of the 2025 preprint are explicit that their findings do not establish that these drugs cause neuroinflammation on their own [8,13]; they instead raise a plausible, hypothesis-generating concern that exogenous GLP-1R agonism could amplify symptoms in patients with an already-inflamed or antibody-primed area postrema, including those with undiagnosed AQP4-IgG seropositivity.

A subsequently published case report offers one, and currently only one, independent data point consistent with this concern: a 29-year-old woman developed a seronegative NMOSD-like syndrome, including longitudinally extensive transverse myelitis and an area postrema lesion, temporally associated with semaglutide use for weight loss [9]. This single case cannot establish causation. Temporal association in one patient is compatible with coincidence, and the patient’s seronegative status makes the AQP4-specific mechanism proposed in Section 4 an imperfect fit for this particular report. It is nonetheless a data point independent of the original conference-presented cohort, and, taken together with the preclinical signal, it supports treating this as an area meriting active pharmacovigilance rather than one to dismiss outright.

Applying a structured causality framework to this case underscores why caution, rather than either dismissal or over-interpretation, is the appropriate stance. Using WHO-UMC categories [26], the report is best classified as “possible”: there is a plausible temporal relationship and a biologically coherent mechanism connecting exogenous GLP-1R agonism to area postrema pathology, but no rechallenge data, no dose–response information, and, given the patient’s seronegative status, an imperfect mechanistic fit with the AQP4-specific pathway proposed in Section 4; alternative explanations, including a coincidental, medication-independent seronegative NMOSD-spectrum presentation, cannot be excluded. Applying the Naranjo adverse drug reaction probability scale [27] yields a score of 2, corresponding to a similarly intermediate “possible” rather than “probable” adverse drug reaction, since criteria such as rechallenge, de-challenge, and a clear dose–response relationship are not met by a single case report. This case-level signal is best complemented, going forward, by disproportionality analyses of spontaneous-reporting pharmacovigilance databases such as the FDA Adverse Event Reporting System (FAERS), which can detect delayed or rare adverse drug reactions across large exposed populations that a single case cannot; to our knowledge, no such formal signal-mining analysis for NMOSD-spectrum presentations following GLP-1 receptor agonist exposure has yet been published, and we identify this as a concrete, actionable next step in Section 9.

A related but mechanistically distinct consideration is the well-documented tendency of GLP-1 receptor agonists to cause nausea at treatment initiation in the general population, reported in large real-world and pediatric/adolescent cohorts independent of any demyelinating disease [28,29,30]. This common, dose-titration-related nausea is generally attributed to peripheral effects on gastric emptying and direct gut–vagal signaling rather than to any central neuroinflammatory process, and it typically attenuates with continued, gradually up-titrated dosing. It is nonetheless worth considering whether at least part of this early nausea reflects a direct, acute effect on the area postrema itself, driven by a sharp rise in circulating and CSF GLP-1 at treatment initiation acting on the chemoreceptor trigger zone’s native GLP-1 receptors (Section 4.1), rather than a purely digestive mechanism, and notably without requiring any dose-dependent astrocytic injury of the kind proposed in Section 4 for NMOSD. If so, ordinary GLP-1RA-associated nausea and the rarer, hypothesis-generating NMOSD-spectrum signal discussed above may represent two ends of a spectrum of area-postrema GLP-1 sensitivity, physiological and self-limited at one end, pathologically amplified by antibody- and complement-mediated injury at the other, rather than two unrelated phenomena. This distinction is speculative and has not been directly tested, but it offers a testable extension of the hypothesis in Section 4 and is noted here as a direction warranting dedicated investigation.

Section 4 authors also reported that satralizumab, an approved interleukin-6-receptor-directed NMOSD therapy, appeared to reduce GLP-1 levels in gut tissue in their model. This is a preliminary observation, raised here only to illustrate the hypothesis’s therapeutic breadth, not as evidence of an established drug interaction [8]. Until further data are available, clinicians evaluating new or atypical intractable nausea and vomiting in a patient recently started on a GLP-1 receptor agonist may reasonably keep NMOSD and related disorders in the differential, particularly if other atypical neurological features are present, rather than defaulting reflexively to a medication side-effect explanation. This is a hypothesis-generating signal rather than an established interaction and should be framed as such in patient counseling and in the literature.

## 8. Limitations

This review has several limitations that should be considered alongside its conclusions.

First, and most importantly, the central mechanistic claim organizing this review, that GLP-1/GLP-1R signaling drives area postrema neuronal hyperexcitability in NMOSD, rests on a single research group’s data, presented as a conference abstract and subsequently posted as a preprint that has not yet completed formal peer review [8,13]. We have made an explicit effort throughout to flag this evidentiary status rather than present the finding as an established mechanism, but readers should treat every downstream implication—biomarker use, therapeutic targeting, and the cautionary signal regarding exogenous GLP-1 receptor agonists—as hypothesis-generating pending independent replication in a separate cohort.

Second, this is a narrative rather than a systematic review. Sources were identified through directed literature and preprint-server searching around area postrema syndrome, NMOSD, MOGAD, GFAP-astrocytopathy, and GLP-1/GLP-1R, rather than through a pre-registered protocol, dual independent screening, or formal risk-of-bias assessment, and the review does not claim to be exhaustive. Methodological details are reported in Section Materials and Methods rather than as a separate, quantitatively pooled Results section, consistent with recommended narrative-review reporting practice [10].

Third, the cross-disorder frequency comparisons in Section 5 draw on a heterogeneous set of retrospectives, largely single-center cohorts that differ in antibody-testing platform, imaging protocol, case ascertainment, and population ethnicity, factors discussed in Section 5.1 that limit how precisely any individual percentage figure can be generalized.

Fourth, the case report discussed in Section 7.4 is a single, seronegative case and should not be over-interpreted as establishing a causal drug–disease interaction; it is presented as one early, independent signal alongside the preclinical data, not as confirmatory evidence.

Fifth, this review addresses adult APS; pediatric APS is not treated as a separate topic (Section 5.2). Because MOGAD, not NMOSD, is the more common host disease in children, and the pediatric differential diagnosis (e.g., cyclic vomiting syndrome, mitochondrial disease) differs from the adult one, the findings and recommendations in this review should not be assumed to generalize to pediatric patients without dedicated study.

Finally, because this is a rapidly evolving area, we updated the literature search shortly before submission (Section 2). Given the pace of publication in this space, readers should independently verify whether additional primary data, particularly a peer-reviewed version of the GLP-1/GLP-1R dataset, have since become available.

## 9. Future Directions

Several lines of investigation follow directly from the gaps identified above.

First, the GLP-1/GLP-1R finding requires independent replication in additional NMOSD cohorts outside the original center, ideally with serial CSF and serum sampling to establish its behavior as a state biomarker of attack activity and treatment response, and with pre-specified thresholds that could support its use in ambiguous presentations.

Second, whether the same axis is active in MOGAD- or GFAP-astrocytopathy-associated APS, or whether their distinct immunopathology and more diffuse lesion pattern (Table 1) reflect a mechanistically distinct process, has not yet been tested and would meaningfully clarify whether GLP-1-directed approaches could generalize beyond AQP4-positive disease.

Third, pharmacovigilance and real-world cohort studies examining GLP-1 receptor agonist exposure alongside NMOSD relapse or APS incidence, together with continued case-level reporting such as the seronegative presentation described in Section 7.4, would help move the current cautionary signal from plausible to evidence-based and could inform prescribing guidance for patients with known demyelinating disease.

Fourth, human proof-of-concept studies of GLP-1R antagonism as adjunctive symptomatic therapy for acute APS attacks represent a logical, mechanistically motivated next step, potentially alongside standard antiemetic regimens in early-phase trials.

Fifth, prospective, multicenter studies applying a single harmonized APS definition and imaging protocol simultaneously across NMOSD, MOGAD, and GFAP-astrocytopathy cohorts would resolve much of the cross-study heterogeneity noted in Section 5.1 and Table 1 and would allow the frequency and lesion-extent comparisons in this review to be tested directly rather than assembled retrospectively from separate studies.

Sixth, and more fundamentally, the GLP-1/GLP-1R hypothesis should be stated in a form that is explicitly testable and falsifiable. If the hypothesis is correct, elevated GLP-1/GLP-1R should track specifically with APS activity within AQP4-IgG-positive NMOSD rather than with NMOSD activity generally, and should not be similarly elevated, or should be elevated to a materially lesser degree, in MOGAD- or GFAP-astrocytopathy-associated APS, given the more diffuse, less AQP4/complement-dependent injury in those disorders (Section 5); replication studies should pre-specify sensitivity and specificity thresholds for GLP-1/GLP-1R as an APS biomarker against the Shosha et al. clinical criteria, rather than reporting group-level differences alone. The hypothesis also generates a population-level prediction: if exogenous GLP-1R agonism meaningfully precipitates NMOSD-spectrum disease in antibody-primed individuals, the substantial recent expansion in GLP-1 receptor agonist prescribing for diabetes and obesity would be expected to associate with a detectable rise in NMOSD or APS incidence at a population level. To our knowledge no such signal has been reported to date, and while the rarity of NMOSD and the short interval since GLP-1RA use became widespread limit how much weight can currently be placed on this absence of evidence, future pharmacoepidemiological studies (above) should explicitly test for it, since a sustained absence of such a signal would meaningfully constrain, rather than support, the cautionary hypothesis discussed in Section 7.4.

## 10. Conclusions

Area postrema syndrome sits at the intersection of anatomy, autoimmunity, and, as tentatively proposed by a single, not-yet-peer-reviewed dataset, endocrinology. The 2025/2026 preprint report of GLP-1/GLP-1R-associated neuronal hyperactivity offers a candidate, hypothesis-generating account of why this particular symptom cluster arises [8], and a plausible, though unproven, frame for understanding why NMOSD, MOGAD, and GFAP-astrocytopathy differ mechanistically as well as in how often and how extensively APS manifests. Independent replication is required before this mechanism can be considered established, or before it should directly inform biomarker development or therapeutic targeting.

What is better established, and does not depend on the GLP-1 hypothesis, is the well-documented tendency for APS to be misread as gastrointestinal disease. This underscores a durable clinical message independent of any single mechanistic finding: intractable, unexplained nausea, vomiting, or hiccups lasting ≥48 h, particularly when accompanied by an unrevealing gastrointestinal evaluation or neurological features, should prompt consideration of neurological and serological evaluation for an antibody-mediated demyelinating disorder before, not after, an extensive gastrointestinal workup.

## Figures and Tables

**Figure 1:**
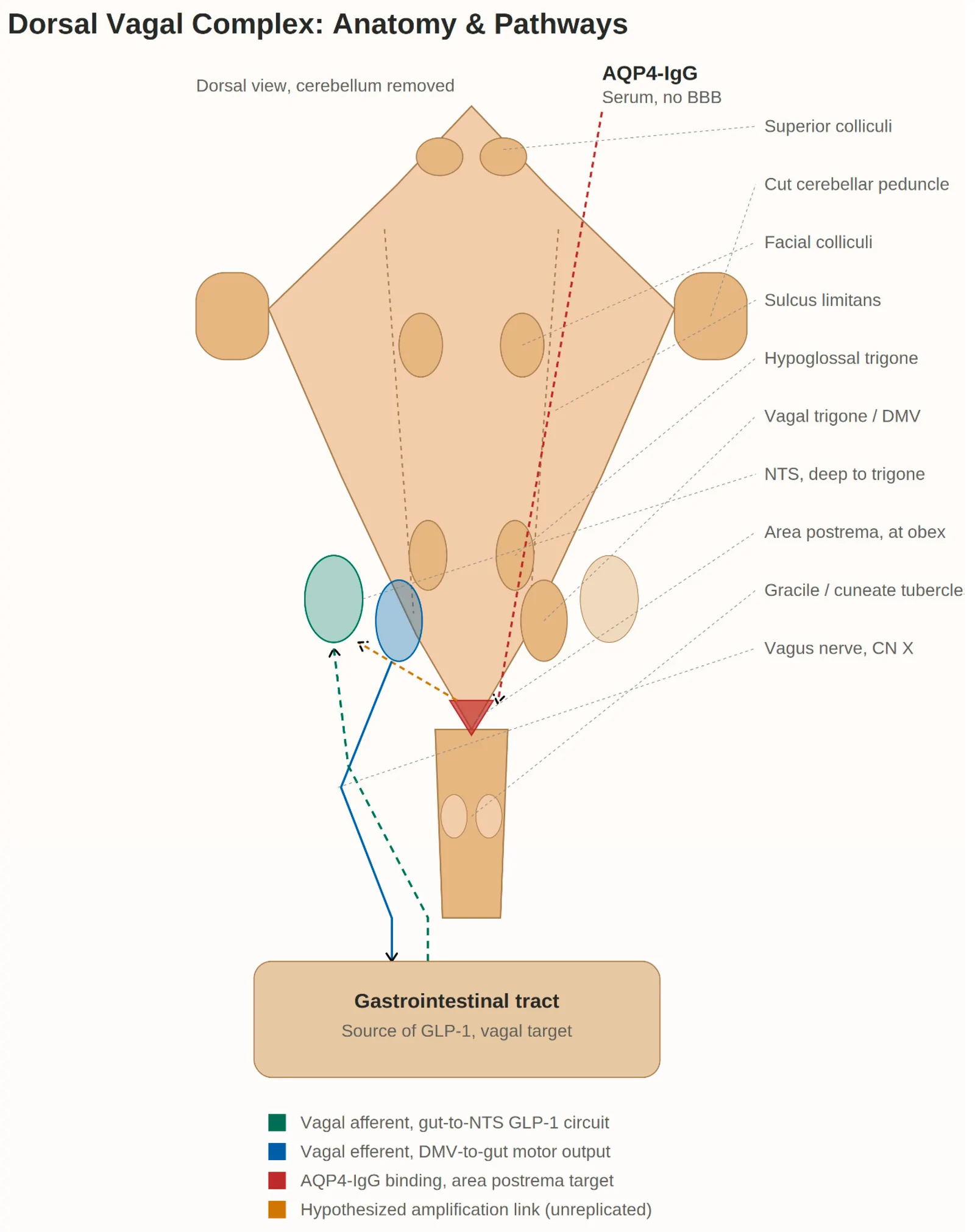
Schematic anatomy of the dorsal vagal complex (area postrema, nucleus tractus solitarius, dorsal motor nucleus of the vagus) and the proposed GLP-1/GLP-1R circuit. Red indicates the established AQP4-IgG/complement-mediated astrocytic injury pathway (Section 3.1); green indicates physiological gut–vagal GLP-1 signaling (Section 4.1); amber indicates the proposed pathological amplification linking the two (Section 4.2), which remains a single-center, not-yet-independently replicated hypothesis. Schematic representation, not a histological or radiological image.

**Figure 2:**
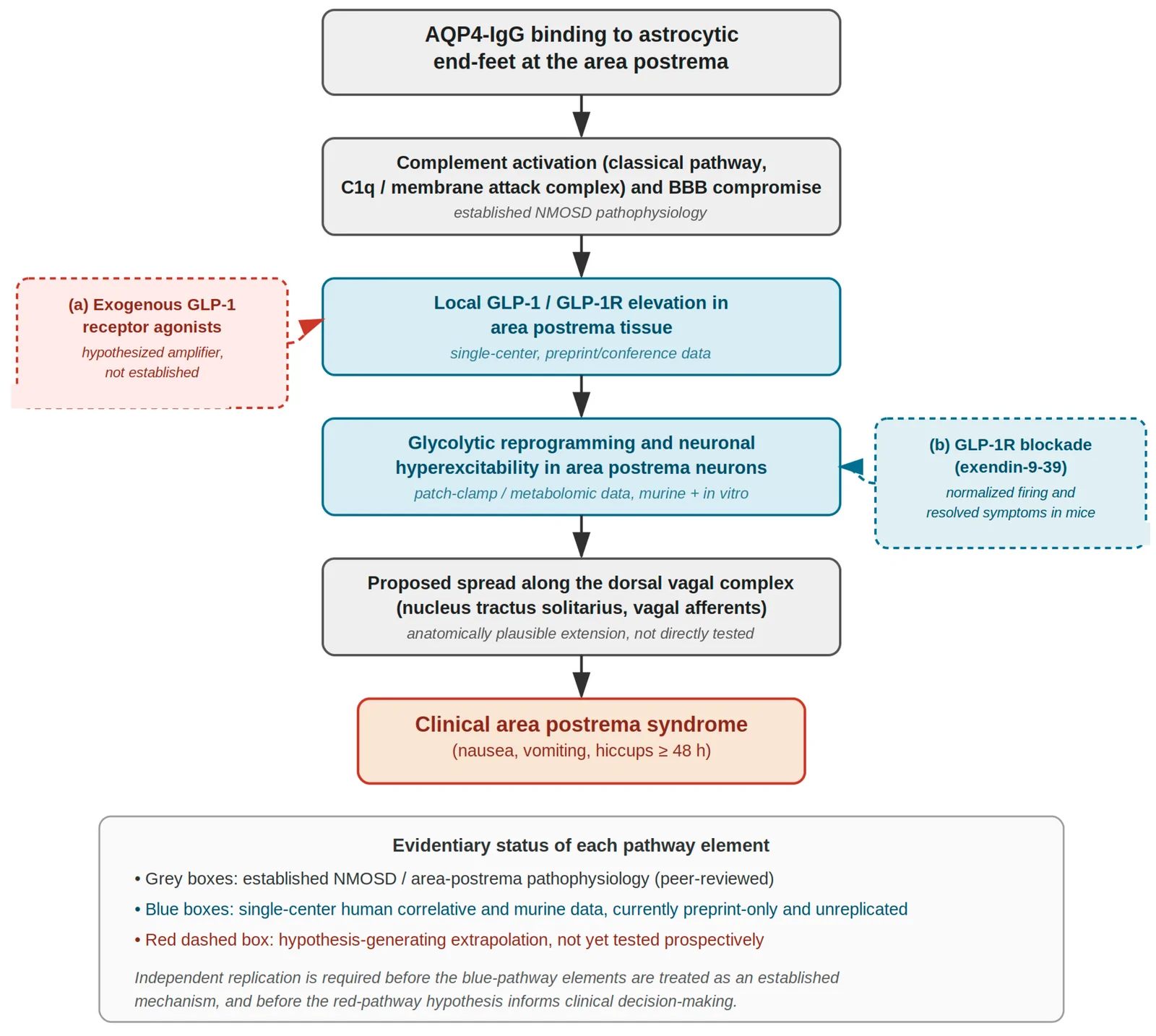
Proposed mechanistic cascade linking AQP4-IgG-mediated area postrema injury to GLP-1/GLP-1R-associated neuronal hyperexcitability and clinical area postrema syndrome. Grey boxes indicate established NMOSD pathophysiology; blue boxes indicate single-center, preprint-only human and murine findings that have not yet been independently replicated; the red dashed box indicates a hypothesis-generating extrapolation that has not been tested prospectively. (a) Hypothesized, not yet established, amplification of this pathway by exogenous GLP-1 receptor agonists. (b) GLP-1R blockade with exendin-(9-39), which normalized neuronal firing and resolved symptoms in animal models. The orange box denotes the resulting clinical syndrome (nausea, vomiting, and/or hiccups), the composite endpoint toward which the upstream pathway proposals converge.

**Figure 3:**
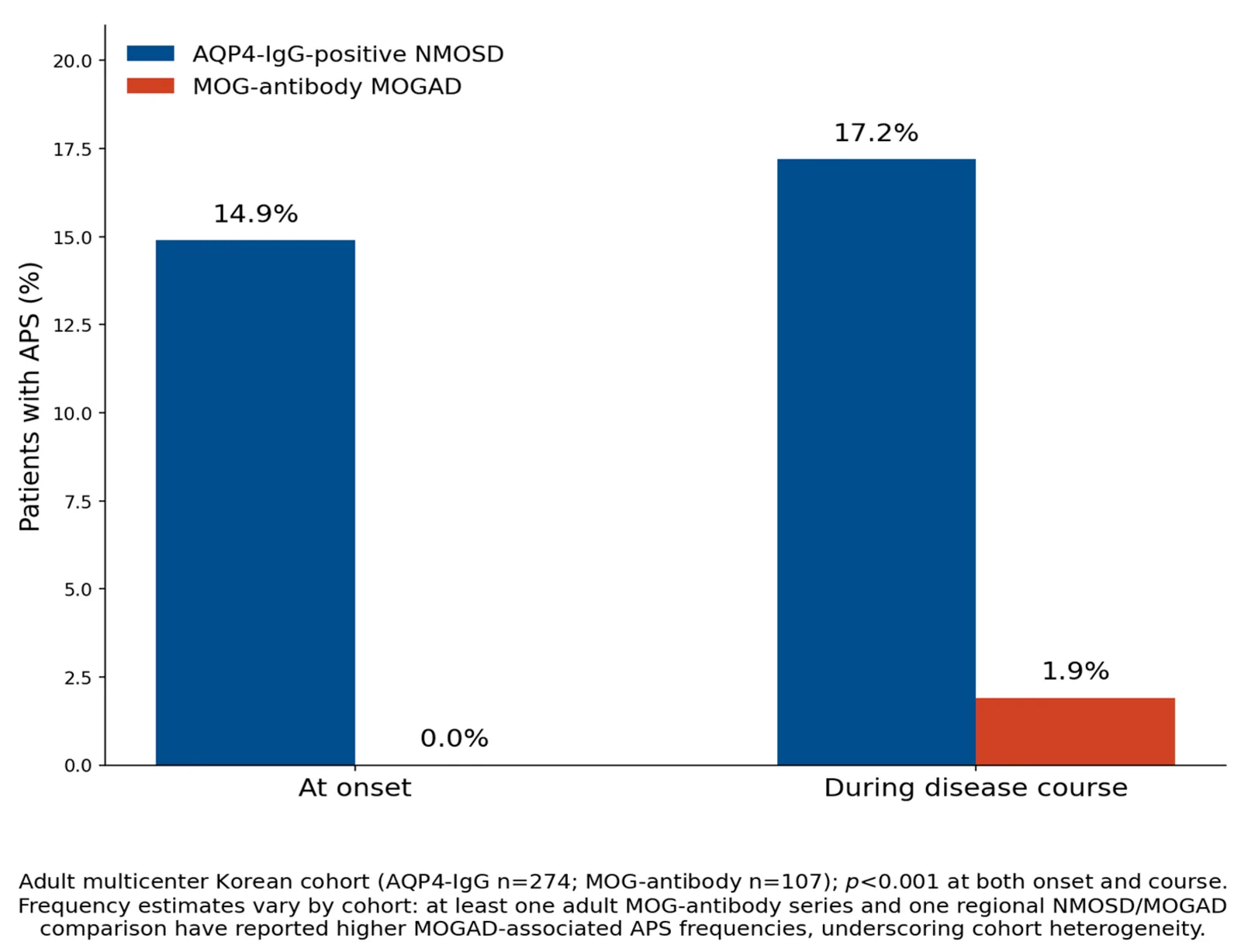
Frequency of area postrema syndrome: AQP4-IgG-positive NMOSD vs. MOG-antibody MOGAD.

**Figure 4:**
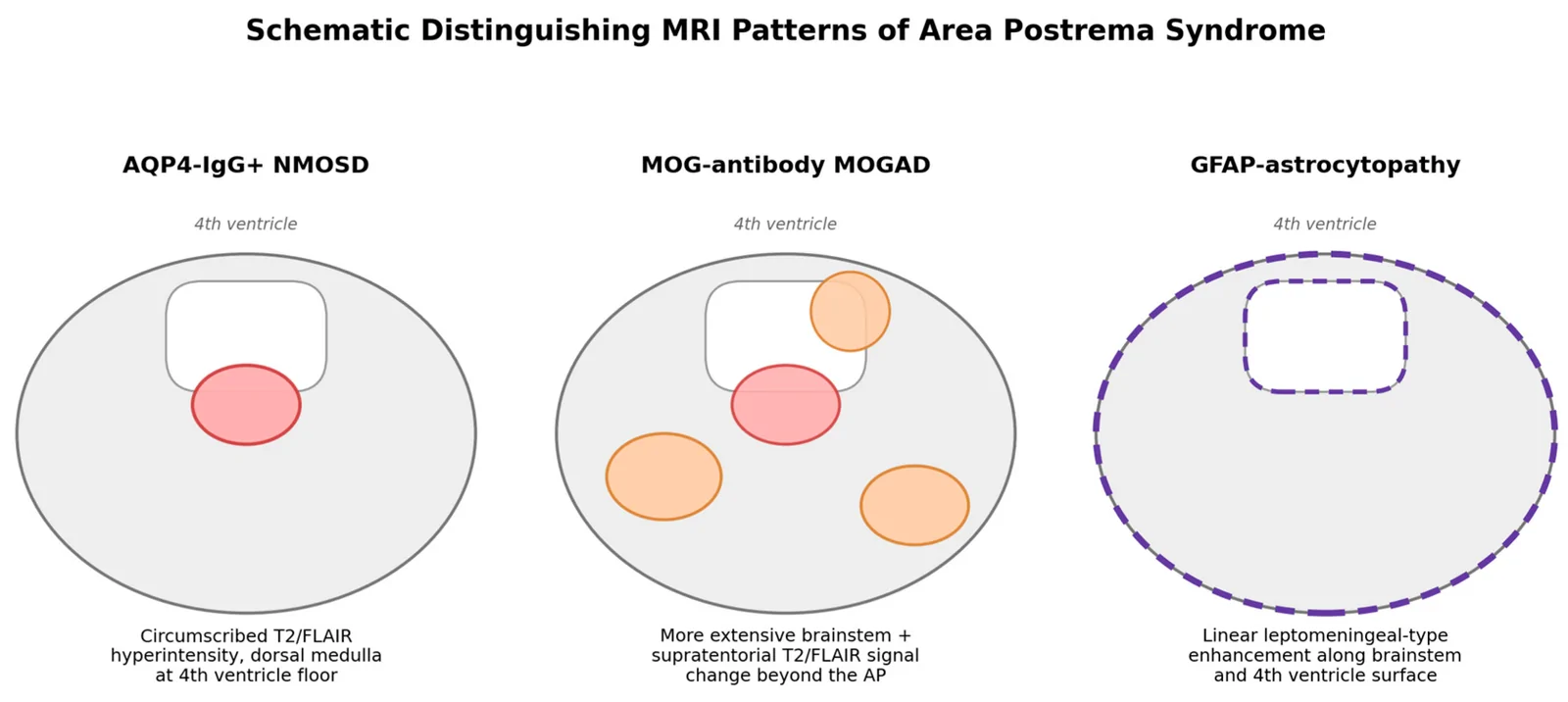
Schematic comparison of the distinguishing MRI patterns of area postrema syndrome across the three disorders (Table 1): a comparatively circumscribed dorsal medullary lesion (red circle) in AQP4-IgG+ NMOSD; more extensive brainstem and supratentorial (orange circles) T2/FLAIR change in MOG-antibody MOGAD, and linear leptomeningeal-type enhancement (purple lines) along the brainstem/fourth ventricle surface in GFAP-astrocytopathy. Schematic representation for illustrative purposes; not derived from a specific patient image, and individual lesions vary in extent within each disorder.

**Table 1:** Comparative summary of area postrema syndrome across the three principal antibody-mediated demyelinating disorders.

Feature	AQP4-IgG+ NMOSD	MOG-Antibody MOGAD	GFAP-Astrocytopathy
APS frequency, onset	~7–15% (higher in some cohorts)	~0–15%; highly cohort-dependent	Uncommon; case-series-level only
APS frequency, disease course	~9–17%	~2–30%; one regional cohort reports higher frequency than NMOSD	Uncommon
Primary antigen/immunopathology	AQP4 (astrocytic water channel); complement-fixing, cytotoxic	MOG (oligodendrocyte/myelin surface); antibody- and T-cell-mediated demyelination	GFAP (intracellular astrocytic filament); T-cell-mediated meningoencephalitis
Typical lesion extent when APS occurs	Comparatively localized dorsal medulla/area postrema	Often more extensive brainstem and supratentorial involvement	Area postrema lesion ± linear leptomeningeal-pattern enhancement
Distinguishing imaging clue	T2/FLAIR ± enhancing dorsal medullary lesion at 4th ventricle floor	Broader brainstem/other brain region T2 signal change	Linear surface enhancement along brainstem/4th ventricle
GLP-1/GLP-1R mechanism demonstrated?	Yes, single-center, preprint (2025/2026)	Not yet studied	Not yet studied
Representative references	[1,2,6,8,13].	[14,15,16,17,18]	[19,20]

## Data Availability

No new data were created or analyzed in this study. Data sharing is not applicable to this article.
